# Development of Functional Lamb Pâté Enriched with Edible Lamb By-Products and Pulse Ingredients: Physicochemical, Nutritional and Safety Evaluation

**DOI:** 10.3390/foods15203647

**Published:** 2026-10-14

**Authors:** Zhuldyz Tokanova, Kumarbek Amirkhanov, Samat Kassymov, Zhanna Assirzhanova, Zhanat Iskakova, Assemgul Baikadamova, Altyngul Khaimuldinova, Alimzhan Imanbayev

**Affiliations:** 1 Department of Food Technologies, Shakarim University, 20 A Glinka Street, Semey 071410, Kazakhstan; judi.93@mail.ru (Z.T.); asirzhanova@shakarim.kz (Z.A.); a.baikadamova@shakarim.kz (A.B.); 2 Department of Food Safety and Technology, Kazakh National Agrarian Research University, 8 Abay Avenue, Almaty 050010, Kazakhstan; lady.iskakova2015@yandex.kz; 3 Department of Standardization, Certification and Metrology, L. N. Gumilyov Eurasian National University, 2 Satpayev Street, Astana 010008, Kazakhstan; akhaymuldinova@bk.ru; 4 Department of Technology and Food Safety, M. Auezov South Kazakhstan State University, 5 Tauke Khan Avenue, Shymkent 160012, Kazakhstan; algo101150@gmail.com

**Keywords:** lamb pâté, edible animal by-products, lamb liver, lamb heart, mung bean, chickpea, hybrid meat products

## Abstract

The growing demand for nutritionally enriched and sustainable meat products has stimulated interest in the utilization of edible animal by-products and plant-based ingredients. The present study aimed to develop lamb pâté formulations containing lamb liver, lamb heart, mung bean, and chickpea and to evaluate their physicochemical, functional–technological, nutritional, microbiological, and contaminant characteristics. Four formulations were prepared, including a control without lamb offal and three experimental formulations containing 13%, 15%, and 17% lamb offal. Mung bean and chickpea were maintained at fixed levels of 7.5% each in all formulations; therefore, the experimental design primarily evaluated the replacement of lean lamb meat with increasing proportions of liver and heart. The effects of lamb offal incorporation on proximate composition, water-holding capacity, water-retention capacity, sensory characteristics, amino acid composition, vitamin and mineral composition, microbiological quality, and heavy metal content were investigated. Among the formulations evaluated, O-2 containing 15% lamb offal showed a favorable combination of physicochemical, water-binding, and sensory characteristics, including a protein content of 26.0%, fat content of 24.9%, moisture content of 62.5%, and the highest water-holding capacity (70.0%). O-2 also received the highest numerical sensory scores, although these were not significantly different from O-1 for several sensory attributes. Amino acid analysis confirmed the presence of essential and non-essential amino acids in the selected O-2 formulation. Compared with the control, O-2 showed numerically higher concentrations of the investigated B-group vitamins and significantly higher concentrations of iron, calcium, and potassium. At the investigated time point, O-2 showed a low total viable count (2.1 × 10^2^ CFU/g), no detectable pathogenic microorganisms, and compliance with the evaluated microbiological criteria; lead and mercury were not detected under the analytical conditions used. Overall, the results indicate that partial replacement of lean lamb meat with edible lamb liver and heart within the fixed pulse-containing formulation can provide pâté with favorable physicochemical, water-binding, sensory, and nutritional characteristics. Further storage studies are required to establish shelf-life stability and microbiological safety throughout storage.

## 1. Introduction

The increasing demand for nutritionally balanced and sustainable food products has stimulated interest in the development of functional meat products enriched with value-added ingredients. Consumers are increasingly seeking foods that not only provide essential nutrients but also contribute to health promotion and sustainable utilization of food resources [1]. In this context, the efficient use of edible animal by-products represents an important strategy for improving resource utilization while enhancing the nutritional value of meat products [2].

Edible animal by-products, including liver, heart, kidneys, and other organs, are recognized as valuable sources of high-quality proteins, essential amino acids, vitamins, and minerals. Lamb liver is particularly rich in iron, vitamin A, riboflavin, niacin, and other micronutrients, whereas lamb heart contains considerable amounts of protein, B-group vitamins, and bioavailable minerals [3,4]. Despite their nutritional advantages, these raw materials remain underutilized in many countries because of limited consumer acceptance and the lack of diversified processed products based on edible offal [5].

Pâté is a widely consumed ready-to-eat meat product characterized by a soft texture, spreadability, and high nutritional value. Traditionally, pâté products are manufactured from liver, meat, fat, and seasonings. In recent years, increasing attention has been devoted to the reformulation of pâté products through the incorporation of functional ingredients aimed at improving nutritional quality, technological properties, and consumer acceptance [6,7]. Several studies have demonstrated that edible animal by-products can be successfully incorporated into pâté formulations, resulting in products with enhanced protein content and micronutrient composition [8].

In parallel with the utilization of animal by-products, plant-based ingredients have attracted considerable interest in the meat industry. Pulses such as chickpea (*Cicer arietinum* L.) and mung bean (*Vigna radiata* L.) are rich sources of proteins, dietary fiber, vitamins, minerals, and bioactive compounds [9,10]. Pulse proteins possess favorable functional properties, including water-binding, emulsifying, and gel-forming capacities, which make them attractive ingredients for hybrid meat products [11]. Previous studies have shown that the incorporation of pulse ingredients may improve moisture retention, texture, nutritional quality, and processing yield of meat products while reducing overall fat content [12].

Chickpea proteins are characterized by a balanced amino acid composition and high nutritional value. Although chickpea proteins are relatively limited in sulfur-containing amino acids, they are rich in lysine and complement animal proteins effectively [13]. Mung bean proteins also exhibit desirable technological properties and contribute to the formation of stable protein matrices in food systems. Their high hydration and emulsifying capacities have been associated with improved water-holding capacity and physicochemical stability of processed meat products [6].

Previous studies have investigated pâté products containing sheep or lamb liver and other edible animal by-products, while pulse ingredients have also been explored separately as plant-based components in hybrid meat products. However, information on pâté formulations combining lamb liver and heart with both mung bean and chickpea remains limited. In particular, the effect of replacing lean lamb meat with increasing proportions of liver and heart within a formulation containing fixed levels of mung bean and chickpea has not been sufficiently characterized. Therefore, the present study aimed to develop lamb pâté formulations containing fixed levels of mung bean and chickpea and increasing proportions of edible lamb liver and heart, and to evaluate their physicochemical, functional–technological, sensory, nutritional, microbiological, and contaminant characteristics. This approach allowed the effects associated with partial replacement of lean lamb meat by edible lamb by-products to be evaluated within a constant pulse-containing formulation.

## 2. Materials and Methods

### 2.1. Raw Materials

**Figure 1:**
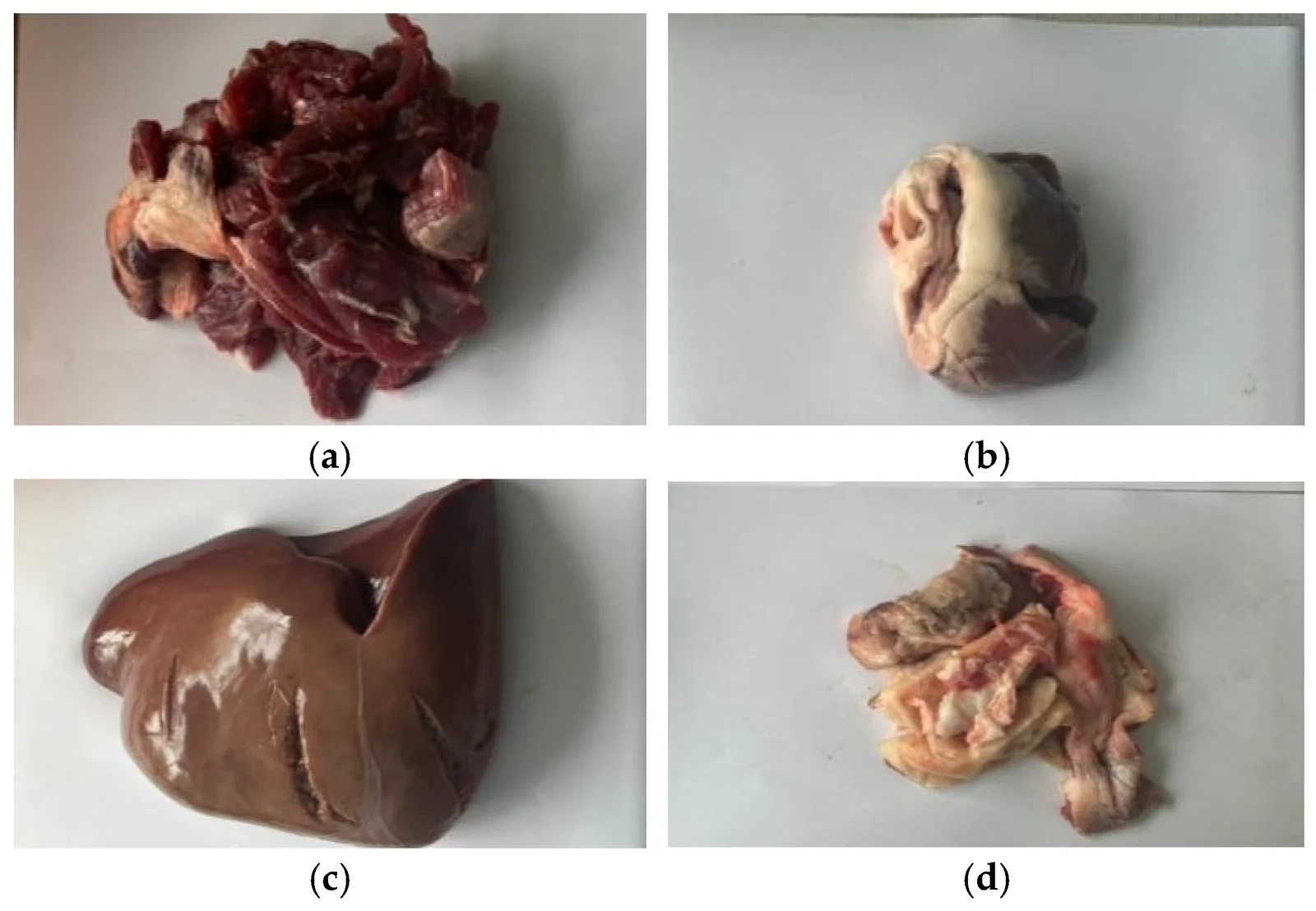
(**a**) Lean lamb meat; (**b**) lamb heart; (**c**) lamb liver; and (**d**) lamb tail fat.

Fresh lean lamb meat, lamb liver, lamb heart, and lamb tail fat were obtained from a local meat-processing facility in Almaty, Kazakhstan (Figure 1). The animal raw materials were transported under refrigerated conditions and maintained at 0–4 °C prior to pâté production. Before processing, the lamb meat, liver, and heart were visually inspected for their suitability for food production. Visible connective tissue and other non-edible portions were removed prior to further processing.

Lamb liver and lamb heart were selected as edible animal by-products for incorporation into the experimental pâté formulations. Lean lamb meat constituted the principal meat component, whereas lamb tail fat was used as the lipid component. The liver and heart were incorporated at different proportions to obtain total offal inclusion levels of 13%, 15%, and 17% in formulations O-1, O-2, and O-3, respectively. Lamb tail fat was maintained at a constant level of 3% in all formulations.

Mung beans (*Vigna radiata* L.) and chickpeas (*Cicer arietinum* L.) were purchased from a local food supplier and inspected for quality before use. Both pulse ingredients were incorporated at 7.5% of the total formulation, corresponding to a combined pulse content of 15%. Additional ingredients included onion, carrot, sodium chloride, garlic, black pepper, coriander, cumin, and paprika. All ingredients used in pâté production were food-grade and were handled under hygienic conditions prior to processing.

### 2.2. Formulation of Experimental Pâté Samples

The formulation of the control and experimental pâté samples is presented in Table 1. The control sample was prepared without the addition of lamb offal, whereas the experimental formulations contained either 13%, 15%, or 17% lamb offal.

Lamb liver and lamb heart were incorporated as valuable sources of animal protein, essential amino acids, minerals, and vitamins. Mung bean and chickpea were included as plant-based ingredients to improve the nutritional profile and functional properties of the product.

Four pâté formulations were developed: a control formulation without lamb offal and three experimental formulations containing different proportions of lamb liver and heart (O-1, O-2, and O-3). The formulations differed in the proportions of lean lamb meat, liver, and heart, while the amounts of the remaining ingredients were kept constant (Table 1). All formulations were prepared under identical processing conditions to ensure comparability among the experimental samples.

The formulations of the control and experimental pâté samples are presented in Table 1.

Mung bean and chickpea were included as plant-based components to complement the animal-derived ingredients and contribute to the nutritional and technological characteristics of the pâté. Both ingredients were incorporated at equal fixed levels of 7.5% (*w*/*w*), resulting in a total pulse content of 15% in each formulation. These levels were selected as formulation proportions and were kept constant across all treatments to allow the experimental comparison to focus on the replacement of lamb meat with increasing proportions of edible lamb by-products (liver and heart). Therefore, the present study was not designed to independently evaluate the effect of pulse incorporation or pulse–offal interactions.

The control formulation contained no edible lamb by-products but retained the same fixed levels of mung bean and chickpea as the experimental formulations. It was therefore used as a reference formulation for evaluating changes associated with the replacement of lamb meat by increasing proportions of liver and heart within the defined formulation system. A lamb-meat-only formulation without pulse ingredients was not included because the pulse-containing matrix was maintained as a constant component of the experimental design. Consequently, the present study does not allow the independent effects of pulse incorporation to be distinguished from those of the overall formulation matrix, and this is acknowledged as a limitation of the study.

### 2.3. Preparation and Processing of Lamb Pâté

Lamb meat, liver, and heart were trimmed of visible connective tissue and cut into small pieces. Mung beans and chickpeas were cooked for 15 min prior to incorporation into the pâté formulations. All ingredients were weighed according to the formulations presented in Table 1.

The ingredients were added sequentially during processing. The lamb meat, liver, heart, and lamb tail fat were added first, followed by the cooked mung beans and chickpeas, vegetables and other supplementary ingredients, and finally the dry ingredients, including salt and spices. The lamb tail fat was incorporated directly with the animal raw materials without separate pre-treatment. All ingredients were then processed together to obtain a homogeneous, finely comminuted pâté mass. Processing was performed using Royal Catering equipment (1500 W, 50 kg capacity, Expondo, Berlin, Germany). The same ingredient preparation and processing procedure was applied to all formulations.

**Figure 2:**
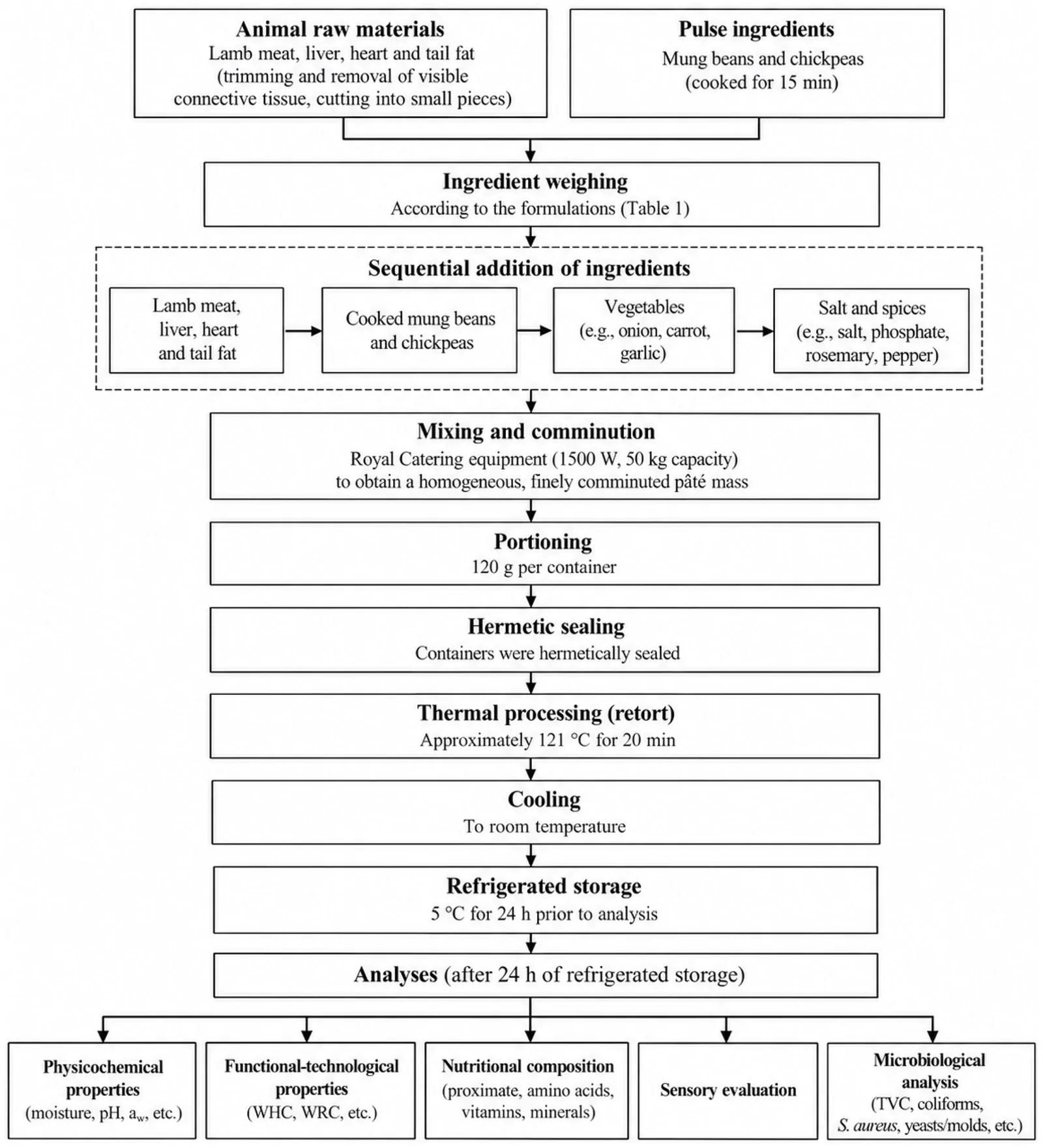
Technological flow diagram for the production of lamb pâté containing edible lamb by-products and pulse ingredients.

The prepared pâté mass was portioned into 120 g containers and hermetically sealed. The sealed products were thermally processed in a retort at approximately 121 °C for 20 min. Following thermal processing, the products were cooled to room temperature and stored under refrigerated conditions at 5 °C for 24 h prior to analysis. All formulations were manufactured using the same technological procedure and processing conditions, differing only in the proportions of lean lamb meat, liver, and heart, while the remaining ingredients were maintained at constant levels. The overall technological process used for the production and subsequent analysis of the pâté formulations is summarized in Figure 2.

### 2.4. Physicochemical Analysis

The proximate composition of the pâté samples, including protein, fat, and moisture contents, was determined using a MultiScan Series 3000 near-infrared (NIR) analyzer (serial No. 2786, Next Instruments, Prestons, NSW, Australia), calibrated for meat products according to the manufacturer’s instructions. Prior to analysis, samples were homogenized to obtain a uniform consistency.

The pH of each sample was measured at 20 ± 2 °C using a calibrated digital pH meter. Approximately 10 g of homogenized pâté was mixed with 100 mL of distilled water before measurement.

Water activity (aw) was determined using a calibrated water activity meter at 25 °C after equilibration of the samples.

Water-holding capacity (WHC) was determined using the pressing method. Approximately 0.3 g of sample was placed between filter papers under a standardized load, and WHC was calculated according to Equation (1).

WHC (%) = [(m_1_ − m_2_)/m_1_] × 100 (1)

where m_1_ is the initial mass of the sample (g), and m_2_ is the mass of the sample after pressing (g).

Water-retention capacity (WRC Index) was evaluated using the filter-paper method based on the ratio between the sample area and the released moisture area. The WRC Index was calculated using Equation (2):

WRC Index = (S_1_/S_2_) × 100 (2)

where S_1_ is the total area occupied by the sample, and S_2_ is the area of the released moisture spot.

The calculated values represent a relative retention index rather than moisture percentage.

All physicochemical and functional–technological measurements were performed in triplicate (*n* = 3), and the results were expressed as mean ± standard deviation.

### 2.5. Amino Acid Analysis

The amino acid composition of the control and selected O-2 formulations was determined according to GOST R 55569-2013 [14] using capillary electrophoresis on a Capel-105M capillary electrophoresis system (Lumex Instruments, St. Petersburg, Russia). The analyses were performed at the Research Laboratory for Quality and Safety Assessment of Food Products, Almaty Technological University (Almaty, Kazakhstan).

Following sample preparation in accordance with GOST R 55569-2013, amino acids were separated by capillary electrophoresis at 30 °C using an applied voltage of 25 kV and detected at a wavelength of 254 nm. The analytical stage duration was 959 s. Amino acids were identified from their electrophoretic migration profiles and quantified using absolute calibration with Elforun software 3.2.2. The results were expressed as mass fraction (%).

### 2.6. Vitamin and Mineral Analysis

Vitamin and mineral contents were determined in the control and selected O-2 formulations at the Research Laboratory for Quality and Safety Assessment of Food Products, Almaty Technological University (Almaty, Kazakhstan).

B-group vitamins were determined by capillary electrophoresis using a Kapel-105M capillary electrophoresis system (St. Petersburg, Russia) in accordance with GOST 31483-2012 [15]. Sample preparation and analytical procedures were performed according to the requirements of the standardized method.

Potassium (K), calcium (Ca), and iron (Fe) were determined according to GOST R 55484-2013 [16], GOST R 55573-2013 [17], and GOST 30178-96 [18], respectively. Sample preparation, calibration, and analytical procedures for mineral determination were performed in accordance with the respective standardized methods. Vitamin and mineral contents were expressed as mg/100 g of product.

### 2.7. Heavy Metal Analysis

Lead (Pb) and mercury (Hg) were determined in the control and selected O-2 formulations. Lead was determined according to GOST 30178-96 [18] using flame atomic absorption spectrometry on a KVANT-2M1 atomic absorption spectrometer (Cortec, Moscow, Russia). Samples were prepared by mineralization in accordance with the standardized method, and Pb concentration was determined in the resulting mineralized solution. Mercury was determined according to GOST 26927-86 [19]. Sample preparation and analytical procedures for Hg determination were performed in accordance with the requirements of the standardized method.

### 2.8. Microbiological Analysis

Microbiological safety was evaluated for the selected formulation (O-2) according to the requirements of TR CU 021/2011 “On Food Safety” [20]. The analyses were performed at the Research Laboratory for Quality and Safety Assessment of Food Products, Almaty Technological University (Almaty, Kazakhstan), using standardized microbiological methods. Total viable count (TVC) was determined according to GOST 10444.15-94 [21]; coliform bacteria according to GOST 31747-2012 [22]; yeasts and molds according to GOST 10444.12-2013 [23]; *Staphylococcus aureus* according to GOST 31746-2012 [24]; *Salmonella* spp. according to GOST 31659-2012 [25]; and *Escherichia coli* according to GOST 30726-2001 [26]. The obtained microbiological results were evaluated against the applicable requirements of TR CU 021/2011 [20].

### 2.9. Sensory Evaluation

Sensory evaluation was conducted using a semi-trained panel consisting of 10 adult participants aged 22–50 years. The panelists evaluated the four pâté formulations (control, O-1, O-2, and O-3) for appearance, color, texture, flavor, and overall acceptability using a 9-point hedonic scale, where 1 represented “dislike extremely” and 9 represented “like extremely”. The samples were presented to the panelists in randomized order under identical evaluation conditions to minimize potential order bias. Sensory scores were subsequently subjected to statistical analysis to determine differences among the formulations. All participants provided informed consent prior to participation in the sensory evaluation. The sensory evaluation was conducted under the ethics exemption described in the Institutional Review Board Statement.

### 2.10. Statistical Analysis

Statistical analyses were performed using SPSS software (version 26.0; IBM Corp., Armonk, NY, USA). The original, untransformed data were used for statistical analysis. Results are presented as mean ± standard deviation (SD). Normality of the data distribution was assessed using the Shapiro–Wilk test, and homogeneity of variances was evaluated using Levene’s test. Differences among the formulations were assessed using one-way analysis of variance (ANOVA), followed by Tukey’s honestly significant difference (HSD) post hoc test for multiple comparisons, where applicable. Differences were considered statistically significant at *p* < 0.05. Different superscript letters within the same row indicate statistically significant differences among formulations.

## 3. Results

### 3.1. Effect of Lamb Offal Incorporation on Chemical Composition and Physicochemical Properties

**Figure 3:**
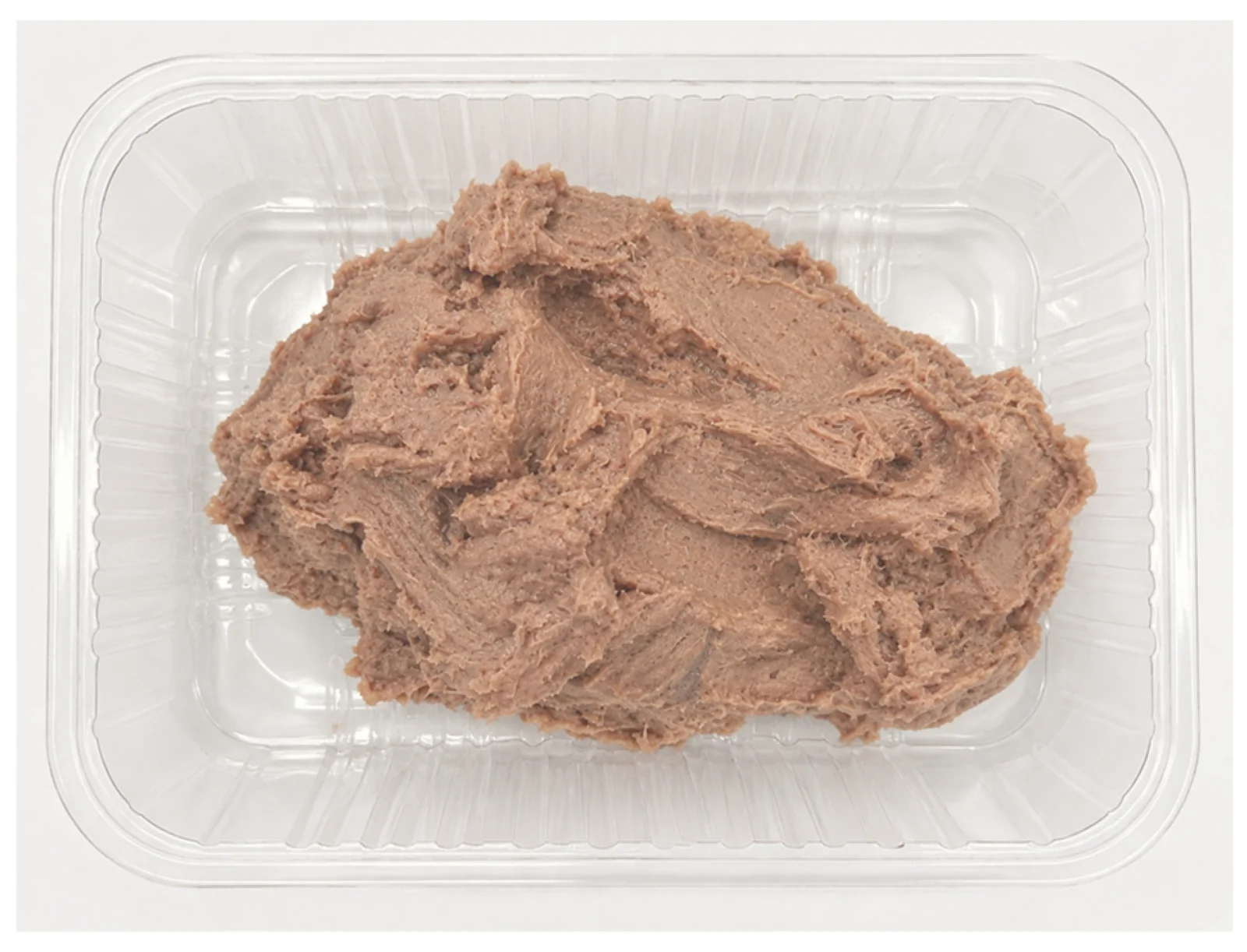
Representative appearance of the developed lamb pâté after thermal processing.

The proximate composition and physicochemical characteristics of the developed pâté samples are presented in Table 2. The visual appearance of the developed pâté after thermal processing is shown in Figure 3.

Significant differences (*p* < 0.05) were observed among the formulations for protein content, fat content, and pH, whereas moisture content and water activity (aw) did not differ significantly (*p* > 0.05). The individual replicate values for these parameters are provided in Supplementary Table S1.

Protein content ranged from 26.0% to 28.7%, with the control formulation showing the highest value. Fat content ranged from 23.3% to 25.8%, whereas moisture content varied from 61.0% to 62.5%. These differences reflect the changes in the relative proportions of lean lamb meat and edible lamb by-products among the formulations, since the levels of mung bean and chickpea were kept constant.

The composition observed in the present study is consistent with the general compositional characteristics reported for pâté-type meat products, although direct numerical comparisons should be interpreted cautiously because pâté formulations differ substantially in their proportions of meat, liver, fat, water, and other ingredients [13]. For example, Amaral et al. investigated lamb pâté containing lamb blood, liver, meat fragments, lard, and water and demonstrated that formulation and storage conditions influenced physicochemical characteristics, including moisture [5]. In the present study, however, the relatively narrow ranges of protein, fat, and moisture indicate that incorporation of lamb liver and heart at the investigated levels maintained the overall proximate composition within a relatively similar range across formulations.

The pH values differed significantly among the pâté formulations (*p* < 0.05). The control formulation showed a pH of 5.79 ± 0.08, whereas substantially lower values were observed for O-1 (4.89 ± 0.06), O-2 (4.90 ± 0.05), and O-3 (4.90 ± 0.07). The three offal-containing formulations showed very similar pH values and belonged to the same statistical group, while the control differed significantly from all experimental formulations. These results indicate that the formulation changes associated with the partial replacement of lean lamb meat by liver and heart were accompanied by a decrease in pH; however, the similar pH values of O-1, O-2, and O-3 indicate that increasing the offal level from 13% to 17% did not produce a further measurable decrease in pH [13].

Water activity values were consistently high in all formulations, which is characteristic of pâté products due to their elevated moisture content and semi-solid structure [27]. High aw values are commonly observed in liver pâtés and other spreadable meat products and are primarily associated with the presence of free water within the protein matrix. Nevertheless, water activity alone does not determine product safety, as microbiological stability is also influenced by thermal treatment, formulation composition, and storage conditions.

Among the investigated formulations, sample O-2 containing 15% lamb offal demonstrated the most balanced physicochemical profile. This sample combined relatively high protein content, moderate fat concentration, improved moisture retention, and stable pH characteristics [6,10]. Therefore, O-2 was selected for further evaluation based on its overall physicochemical performance among the formulations tested.

### 3.2. Functional–Technological Properties of Lamb Pâté Samples

Water-holding capacity (WHC) and water-retention capacity (WRC) are important technological indicators influencing the texture, juiciness, processing yield, and overall quality of meat products. As shown in Table 3, significant differences in both parameters were observed among the formulations (*p* < 0.05). Among the investigated formulations, O-2 exhibited the highest WHC (70.0 ± 3.94%) and WRC index (300.0 ± 10.4), indicating superior water-retention properties compared with the other experimental formulations.

The higher water-binding capacity observed in O-2 may be associated with the specific balance between lean lamb meat and edible lamb by-products in this formulation and their interactions within the protein matrix. Mung bean and chickpea proteins may have contributed to the overall water-binding properties of all formulations because of their hydration capacity; however, as their concentrations were kept constant, they cannot independently explain the differences observed among the experimental formulations. Previous studies have similarly shown that the technological properties of meat products can be influenced by both the source and proportion of animal and plant proteins, as these factors affect protein–water interactions and matrix stability [11,12]. The higher WHC and WRC index observed in O-2 therefore suggest that the 15% offal formulation provided a favorable balance of protein components for water retention under the processing conditions used in the present study.

Overall, O-2 demonstrated the highest WHC and WRC index among the experimental formulations, supporting its selection for subsequent nutritional and microbiological evaluation.

### 3.3. Sensory Characteristics

The sensory characteristics of the lamb pâté formulations are presented in Table 4. Significant differences (*p* < 0.05) were observed among the formulations for the evaluated sensory attributes. O-2 received the highest numerical scores for texture (8.8), flavor (8.9), and overall acceptability (8.9). However, O-1 and O-2 shared the same superscript letter for these attributes, indicating that the differences between these two formulations were not statistically significant (*p* > 0.05).

These findings are consistent with previous studies demonstrating that edible animal by-products can be incorporated into pâté formulations while maintaining acceptable sensory characteristics. Amaral et al. [5] reported satisfactory sensory quality of sheep liver pâté prepared with edible slaughter by-products, while Kenenbay et al. [6] demonstrated the successful development of liver pâté containing edible animal by-products. In the present study, the slight decline in sensory scores observed for O-3 (17% offal) compared with O-2 suggests that increasing the proportion of lamb liver and heart beyond the level used in O-2 did not provide additional sensory benefit and may have contributed to a more pronounced organ-meat flavor.

**Figure 4:**
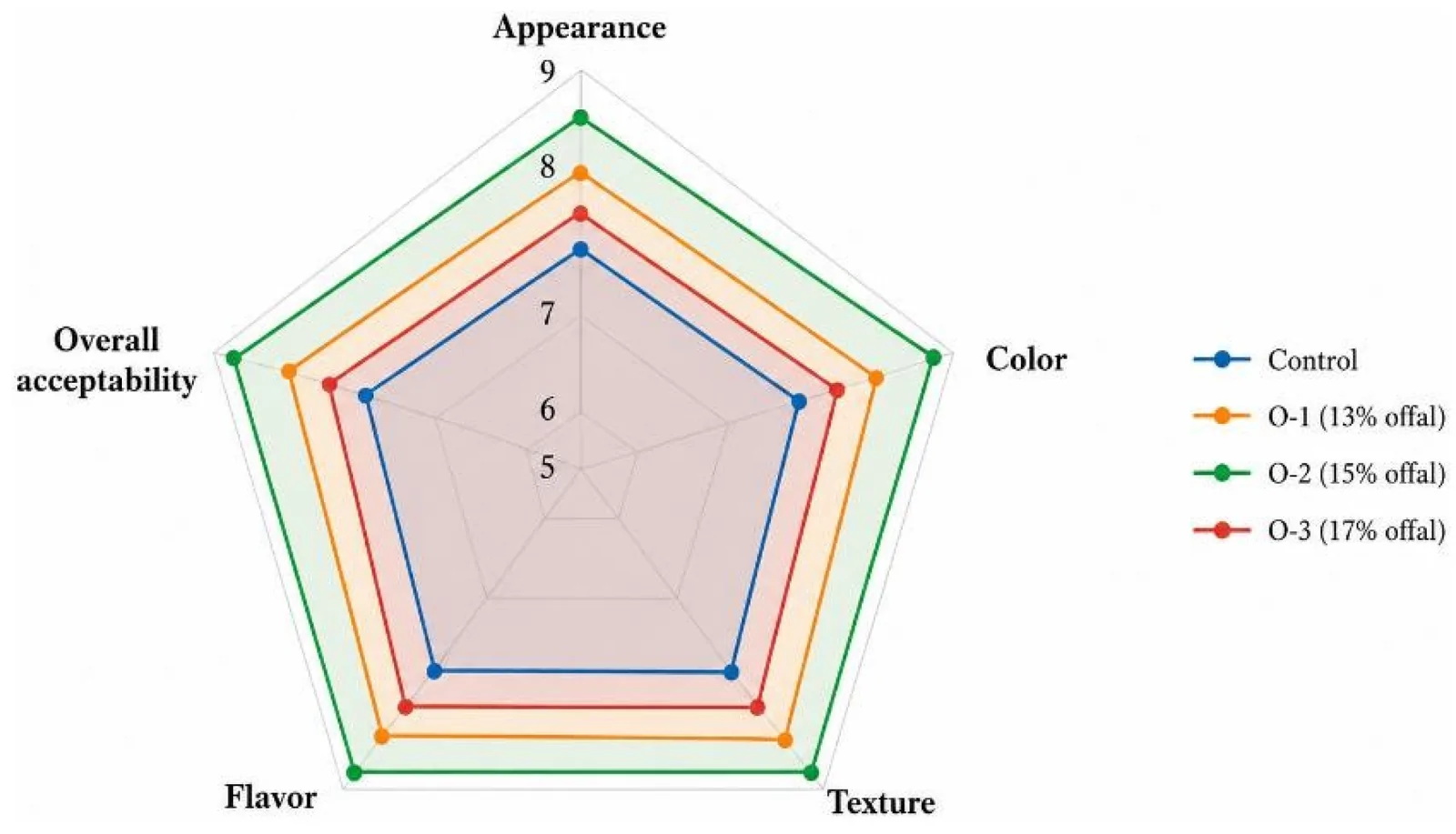
Sensory profiles of the control and experimental lamb pâté formulations based on appearance, color, texture, flavor, and overall acceptability scores.

The radar chart (Figure 4) provides a visual comparison of appearance, color, texture, flavor, and overall acceptability scores obtained using a 9-point hedonic scale. O-2 showed the highest numerical scores across the evaluated sensory attributes. However, O-1 and O-2 were not statistically different for several sensory attributes, and the higher numerical scores of O-2 should therefore not be interpreted as evidence of statistically superior consumer preference. Overall, the sensory results indicate favorable sensory acceptance of the O-2 formulation under the conditions of the present study.

### 3.4. Amino Acid Composition and Protein Quality

Amino acid composition was determined for both the control and selected O-2 formulations (Table 5). Both essential and non-essential amino acids were detected in the two formulations. The amino acid profiles were generally comparable, although numerical differences were observed for individual amino acids.

Compared with the control, O-2 showed numerically higher arginine (0.351 vs. 0.300%) and methionine (0.141 vs. 0.112%) contents, while several other amino acids, including phenylalanine, leucine + isoleucine, valine, proline, threonine, alanine, and glycine, were numerically lower in O-2. Lysine, tyrosine, histidine, and serine showed relatively small numerical differences between the two formulations. As no inferential statistical comparison was performed for these amino acid data, the observed differences were interpreted descriptively and should not be considered statistically significant.

The amino acid profiles of the control and selected O-2 formulations were generally comparable, although numerical differences were observed for individual amino acids. Lysine was detected at 0.305 ± 0.104% in the control and 0.294 ± 0.100% in O-2, while valine contents were 0.535 ± 0.214% and 0.481 ± 0.192%, respectively. Phenylalanine was present at 0.514 ± 0.154% in the control and 0.464 ± 0.139% in O-2. In contrast, O-2 showed numerically higher contents of arginine (0.351 ± 0.140% vs. 0.300 ± 0.120%) and methionine (0.141 ± 0.048% vs. 0.112 ± 0.038%) than the control. These differences are interpreted descriptively because inferential statistical comparisons were not performed for the amino acid data.

Branched-chain amino acids (BCAAs), represented by leucine + isoleucine and valine, were detected in both formulations. Leucine + isoleucine contents were 0.476 ± 0.124% in the control and 0.402 ± 0.104% in O-2, while valine contents were 0.535 ± 0.214% and 0.481 ± 0.192%, respectively. The non-essential amino acid fraction in both formulations included proline, glycine, alanine, serine, and arginine, demonstrating the presence of a broad range of amino acids in both products.

Previous studies have reported favorable amino acid profiles for chickpea and mung bean proteins and have highlighted their potential use as complementary protein sources in composite foods [7,8,28]. However, mung bean and chickpea were maintained at identical concentrations in the control and O-2 formulations in the present study. Therefore, the observed differences in amino acid composition cannot be attributed specifically to the pulse ingredients. Rather, they reflect differences between the overall formulations, particularly the partial replacement of lean lamb meat with lamb liver and heart in O-2.

Protein quality is commonly evaluated using amino acid scoring approaches that consider essential amino acid composition together with protein digestibility [29]. However, amino acid score and protein digestibility were not determined in the present study. Therefore, the results should be interpreted as a compositional comparison of the control and selected O-2 formulations rather than as evidence of superior protein quality.

Overall, amino acid analysis confirmed the presence of both essential and non-essential amino acids in the control and O-2 formulations. The comparison revealed numerical differences in individual amino acids, but these data do not establish an overall improvement in amino acid profile or protein quality in O-2.

### 3.5. Vitamin Composition and Mineral Composition

The vitamin and mineral composition of the control sample and the selected formulation (O-2) is presented in Table 6. Lamb liver and lamb heart are recognized as valuable sources of B-group vitamins and essential minerals, and their incorporation contributed to a numerically improved micronutrient profile of the selected formulation.

The concentrations of thiamine (B1), riboflavin (B2), niacin (B3), and pantothenic acid (B5) were slightly higher in O-2 than in the control sample. Although these differences were relatively small, they are consistent with the naturally high vitamin content of edible lamb by-products, particularly liver and heart [6].

Statistically significant increases (*p* < 0.05) were observed for iron, calcium, and potassium in the selected formulation. The higher iron content is of particular nutritional importance because edible animal by-products represent one of the richest dietary sources of highly bioavailable heme iron. Similar improvements in mineral composition have been reported for meat products enriched with edible by-products [4,27,30,31].

Overall, the selected formulation (O-2) contained both essential and non-essential amino acids. The combination of lamb meat, edible offal, mung bean, and chickpea resulted in a nutritionally relevant amino acid profile.

### 3.6. Microbiological Safety and Heavy Metal Assessment

The microbiological quality and heavy metal assessment of the selected formulation (O-2) are presented in Table 7. All evaluated microbiological indicators complied with the specified requirements of TR CU 021/2011 [20] under the conditions of the study.

The total viable count (2.1 × 10^2^ CFU/g) was substantially lower than the maximum permissible limit (5 × 10^3^ CFU/g). In addition, *Escherichia coli*, coliform bacteria, *Staphylococcus aureus*, and *Salmonella* spp. were not detected, indicating satisfactory microbiological safety of the selected formulation. Similar microbiological profiles have been reported for properly processed liver pâté and other cooked meat products manufactured under hygienic conditions [5,6].

Yeast and mold counts remained low and were within the permissible limits, suggesting minimal fungal contamination. Furthermore, lead (Pb) and mercury (Hg) were not detected in the selected formulation under the analytical conditions used.

Overall, the microbiological and toxicological results confirmed that the selected formulation (O-2) complied with the established food safety requirements under the conditions evaluated.

### 3.7. Selection of the Formulation for Further Analysis

Considering the parameters measured across all four formulations, O-2 showed a favorable overall combination of physicochemical, functional–technological, and sensory characteristics. It exhibited the highest WHC and WRC index and the highest numerical sensory scores among the formulations tested. On this basis, O-2 was selected for subsequent amino acid, micronutrient, microbiological, and contaminant analyses.

Subsequent characterization of O-2 demonstrated the presence of both essential and non-essential amino acids. The formulation also showed numerically higher concentrations of the investigated B-group vitamins and significantly higher concentrations of iron, calcium, and potassium than the control. Microbiological and contaminant analyses further showed that O-2 complied with the evaluated food safety requirements under the conditions of the study. These additional analyses characterized the selected formulation and were not used as comparative criteria for selecting O-2 among the experimental formulations.

Overall, the results indicate that the incorporation of 15% edible lamb by-products combined with pulse ingredients represents a promising approach for the development of lamb pâté with favorable technological and sensory characteristics while also supporting the utilization of edible lamb by-products.

### 3.8. Limitations of the Study

The present study focused on the formulation development and evaluation of physicochemical, functional–technological, nutritional, sensory, and microbiological characteristics of lamb pâté enriched with edible lamb by-products and pulse ingredients. An additional limitation is the absence of a lamb-meat-only control without pulse ingredients; therefore, the independent contribution of mung bean and chickpea cannot be determined from the present experimental design. Shelf-life stability, lipid oxidation, and long-term storage studies were beyond the scope of the present work and should be addressed in future investigations. Further studies should also include texture profile analysis and large-scale consumer acceptance testing to support industrial application of the developed product.

## 4. Discussion

The present study demonstrated that the incorporation of lamb liver and heart in combination with mung bean and chickpea influenced the physicochemical, functional–technological, sensory, and nutritional characteristics of lamb pâté. Among the investigated formulations, O-2 containing 15% lamb offal provided the most favorable overall balance of the parameters evaluated across all four formulations. In particular, O-2 exhibited the highest water-holding capacity (70.0 ± 3.94%) and WRC index (300.0 ± 10.4), together with the highest numerical sensory scores. The decrease in WHC observed in O-3 despite its higher offal content indicates that increasing the proportion of edible offal did not produce a proportional improvement in technological performance. Instead, the results suggest that an appropriate balance between animal and plant-derived ingredients is important for the formation of the pâté matrix.

The pH differed markedly between the control and the offal-containing formulations. The control had a pH of 5.79 ± 0.08, whereas O-1, O-2, and O-3 showed similar values of approximately 4.89–4.90. Thus, the main difference was observed between the control and the experimental formulations, while increasing the proportion of liver and heart from 13% to 17% did not result in further substantial changes in pH. Hamzeh et al. [13] reported that formulation factors, including liver type and fat proportion, can influence the pH and physicochemical characteristics of liver pâté. In the present study, the observed pH difference should therefore be interpreted in relation to the overall formulation change rather than attributed to an individual ingredient [7].

The most pronounced technological differences among the formulations were observed in the water-binding characteristics. WHC increased from 19.23 ± 1.42% in the control to 50.0 ± 2.86% in O-1 and reached its highest value of 70.0 ± 3.94% in O-2, before decreasing to 34.0 ± 2.11% in O-3. The WRC index also reached its highest value in O-2 (300.0 ± 10.4). Because mung bean and chickpea were maintained at identical concentrations in all formulations, the differences in water-binding properties cannot be attributed specifically to pulse incorporation. Instead, the observed variation may reflect differences in the relative proportions of lean lamb meat, liver, and heart and their interactions within the overall pâté matrix. The decrease in WHC observed in O-3 further indicates that the relationship between offal proportion and water retention was not linear. Thus, the higher WHC and WRC values observed in O-2 should be interpreted as characteristics of the overall formulation rather than as effects of any single ingredient. The sensory evaluation provided additional support for the suitability of the O-2 formulation.

O-2 obtained the highest numerical scores for appearance, color, texture, flavor, and overall acceptability. Nevertheless, O-1 and O-2 shared the same statistical group where the same superscript was assigned; consequently, the results should be interpreted as favorable sensory acceptance of O-2 rather than evidence of a statistically superior consumer preference. Amaral et al. [5] similarly demonstrated satisfactory sensory quality of sheep liver pâté prepared using edible slaughter by-products. Kenenbay et al. [6] also reported successful development of liver pâté formulated with edible animal by-products, including beef offal and sheep tail fat. These previous findings, together with the present results, indicate that edible animal by-products can be incorporated into pâté formulations while maintaining acceptable sensory characteristics.

The nutritional composition of O-2 further supports the potential value of edible lamb by-products. Iron concentration was significantly higher in O-2 (8.11 mg/100 g) than in the control (7.80 mg/100 g). Amaral et al. [5] reported approximately 9.0 mg/100 g of iron in sheep liver pâté prepared using edible slaughter by-products. Although differences in formulation and analytical methodology prevent direct quantitative equivalence between the two studies, the results are consistent in demonstrating the potential of sheep and lamb edible by-products to contribute substantial amounts of iron to pâté products.

The mineral findings are also consistent with previous studies of edible animal organs. Seong et al. [3] characterized edible livestock by-products and demonstrated their potential as nutritionally valuable sources of proteins and micronutrients. More specifically for the raw materials relevant to the present work, Biel et al. [4] characterized offal from lamb and other livestock species and demonstrated its considerable nutritional value. In the present study, O-2 contained significantly higher concentrations of Fe, Ca, and K than the control. These results support the potential contribution of lamb liver and heart to the mineral composition of the developed pâté. However, although B-group vitamin concentrations were numerically higher in O-2 than in the control, these differences should not be described as significant enrichment without corresponding statistical evidence.

The amino acid analysis demonstrated the presence of both essential and non-essential amino acids in O-2. The use of animal and pulse-derived ingredients provides different protein sources within the same formulation. Onder et al. [27] demonstrated that chickpea protein isolates contain a range of amino acids and possess relevant nutritional and functional characteristics, while Feng et al. [12] described mung bean protein as an emerging plant protein source with potential food applications. Amaral et al. [5] also reported a nutritionally relevant essential amino acid composition in sheep liver pâté containing edible slaughter by-products. These findings support the nutritional relevance of combining edible lamb organs with pulse ingredients. However, because amino acid analysis in the present study was performed only for O-2, it cannot be concluded that O-2 possessed a superior amino acid profile compared with the control, O-1, or O-3.

The microbiological and contaminant results showed that O-2 complied with the evaluated safety requirements at the time of analysis. The total viable count remained below the specified limit, the investigated indicator and pathogenic microorganisms were not detected at the stated analytical levels, and lead and mercury were not detected. Amaral et al. [5] likewise reported satisfactory microbiological quality of sheep liver pâté prepared with edible slaughter by-products. However, microbiological safety during storage represents a separate issue. Agregán et al. [7] evaluated pork liver pâté throughout storage and demonstrated the importance of shelf-life assessment when determining the stability of pâté products. Since the present study did not include a storage experiment, the microbiological findings should be interpreted as evidence of product quality at the time of analysis and not as evidence of extended shelf-life or microbiological stability during storage.

From a broader perspective, the present results support the valorization of edible lamb by-products through their incorporation into value-added meat products. Toldrá et al. [2] highlighted the potential for value addition through improved utilization of edible meat by-products. Amaral et al. [5] demonstrated this approach specifically in sheep liver pâté, while Kenenbay et al. [6] showed the potential application of edible animal by-products in reformulated liver pâté. The present study extends this approach by combining lamb liver and heart with mung bean and chickpea, thereby integrating the utilization of edible animal by-products with pulse-based ingredients. Such a strategy may contribute to more efficient utilization of animal raw materials while providing technologically and nutritionally relevant food products.

Several limitations of the present study should be considered. Amino acid and microbiological/contaminant analyses were conducted only for O-2, whereas micronutrient composition was compared only between the control and O-2. Therefore, these endpoints cannot be used to establish differences among all experimental formulations. Furthermore, sensory evaluation was performed using a relatively small semi-trained panel (*n* = 10), and shelf-life, lipid oxidation, and instrumental texture were not evaluated. Future studies should investigate the selected formulation throughout refrigerated storage, including microbiological changes, lipid oxidation, physicochemical stability, and instrumental texture, and should confirm sensory acceptance using a larger consumer panel.

## 5. Conclusions

This study demonstrated the feasibility of incorporating lamb liver, lamb heart, mung bean, and chickpea into lamb pâté formulations. Among the investigated formulations, O-2, containing 15% lamb offal (9% liver and 6% heart), provided the most favorable overall combination of physicochemical, water-binding, and sensory characteristics. O-2 exhibited the highest water-holding capacity and WRC index and received the highest numerical sensory scores among the tested formulations.

The selected formulation contained both essential and non-essential amino acids and showed significantly higher concentrations of iron, calcium, and potassium than the control. Microbiological and toxicological analyses showed that O-2 complied with the evaluated food safety requirements under the conditions of the study.

Overall, the results demonstrate the potential of combining edible lamb by-products with pulse ingredients in pâté production and provide an approach for the valorization of underutilized animal raw materials. Further studies should investigate shelf-life stability, lipid oxidation, instrumental texture, and consumer acceptance during refrigerated storage and production scale-up.

## Figures and Tables

**Table 1:** Formulation of lamb meat pâté enriched with lamb offal, mung bean and chickpea (%).

Ingredients (%)	Control	O-1	O-2	O-3
Lean lamb meat	65	52	50	48
Lamb liver	0	8	9	10
Lamb heart	0	5	6	7
Mung bean	7.5	7.5	7.5	7.5
Chickpea	7.5	7.5	7.5	7.5
Onion	5	5	5	5
Carrot	3	3	3	3
Lamb tail fat	3	3	3	3
Water	6	6	6	6
Salt	1.5	1.5	1.5	1.5
Spices	1.5	1.5	1.5	1.5

Values are expressed as percentage of total formulation.

**Table 2:** Proximate composition, pH and water activity (aw) of lamb pâté samples enriched with lamb offal.

Parameter	Control	O-1 (13% Offal)	O-2 (15% Offal)	O-3 (17% Offal)	ANOVA *p*
Protein (%)	28.7 ± 0.66 ^a^	28.2 ± 0.75 ^a^	26.0 ± 0.50 ^b^	26.3 ± 0.65 ^b^	0.0019
Fat (%)	25.8 ± 0.75 ^a^	23.3 ± 0.56 ^b^	24.9 ± 0.70 ^ab^	23.3 ± 0.70 ^b^	0.0045
Moisture (%)	61.7 ± 0.95 ^a^	61.0 ± 0.80 ^a^	62.5 ± 0.90 ^a^	62.4 ± 1.00 ^a^	0.2364
pH	5.79 ± 0.08 ^a^	4.89 ± 0.06 ^b^	4.90 ± 0.05 ^b^	4.90 ± 0.07 ^b^	<0.001
Water activity (aw)	0.9997 ± 0.0003 ^a^	0.9998 ± 0.0002 ^a^	0.9999 ± 0.0001 ^a^	0.9998 ± 0.0002 ^a^	0.7278

Values are presented as mean ± standard deviation (*n* = 3). Different superscript letters within the same row indicate significant differences among formulations according to Tukey’s HSD test (*p* < 0.05). Values sharing the same superscript letter are not significantly different.

**Table 3:** Functional–technological properties of lamb pâté samples.

Sample	WHC (%)	WRC Index
Control (0% offal)	19.23 ± 1.42 ^c^	Not determined
O-1 (13% offal)	50.0 ± 2.86 ^b^	280.0 ± 8.7 ^b^
O-2 (15% offal)	70.0 ± 3.94 ^a^	300.0 ± 10.4 ^a^
O-3 (17% offal)	34.0 ± 2.11 ^c^	285.7 ± 9.5 ^b^

WRC was not determined for the control formulation during the original experimental measurements; therefore, direct comparison of WRC between the control and experimental formulations was not possible. Values are presented as mean ± standard deviation (*n* = 3). Different superscript letters within the WHC column indicate significant differences among formulations (Tukey’s test, *p* < 0.05). Different superscript letters within the WRC column indicate significant differences among the experimental formulations (Tukey’s test, *p* < 0.05). WRC represents a relative water-retention index and is not expressed as a percentage. WRC was not determined for the control formulation.

**Table 4:** Sensory evaluation (*n* = 10).

Parameter	Control	O-1	O-2	O-3
Appearance	7.8 ± 0.5 ^b^	8.2 ± 0.4 ^a^	8.6 ± 0.4 ^a^	8.0 ± 0.5 ^b^
Color	7.7 ± 0.6 ^b^	8.3 ± 0.5 ^a^	8.7 ± 0.4 ^a^	8.1 ± 0.5 ^b^
Texture	7.9 ± 0.5 ^b^	8.4 ± 0.4 ^a^	8.8 ± 0.3 ^a^	8.2 ± 0.4 ^b^
Flavor	7.8 ± 0.6 ^b^	8.5 ± 0.5 ^a^	8.9 ± 0.3 ^a^	8.0 ± 0.5 ^b^
Overall acceptability	7.8 ± 0.5 ^b^	8.4 ± 0.4 ^a^	8.9 ± 0.3 ^a^	8.1 ± 0.4 ^b^

Note: Values are presented as mean ± standard deviation (SD; *n* = 10). Different superscript letters (^a^, ^b^) within the same row indicate statistically significant differences between formulations (*p* < 0.05). Overall, O-2 showed the highest numerical sensory scores among the investigated formulations, indicating favorable sensory acceptance. However, O-1 and O-2 belonged to the same statistical group for the evaluated attributes; therefore, the higher numerical scores of O-2 should not be interpreted as statistically superior sensory acceptance compared with O-1.

**Table 5:** Amino acid composition of lamb pâté enriched with 15% lamb offal.

Amino Acid	Control (%)	O-2 (15% Offal)
Arginine	0.300 ± 0.120	0.351 ± 0.140
Lysine	0.305 ± 0.104	0.294 ± 0.100
Tyrosine	0.230 ± 0.069	0.238 ± 0.071
Phenylalanine	0.514 ± 0.154	0.464 ± 0.139
Histidine	0.155 ± 0.078	0.147 ± 0.074
Leucine + Isoleucine	0.476 ± 0.124	0.402 ± 0.104
Methionine	0.112 ± 0.038	0.141 ± 0.048
Valine	0.535 ± 0.214	0.481 ± 0.192
Proline	0.642 ± 0.167	0.543 ± 0.141
Threonine	0.391 ± 0.156	0.339 ± 0.136
Serine	0.391 ± 0.102	0.351 ± 0.091
Alanine	0.428 ± 0.111	0.351 ± 0.091
Glycine	0.418 ± 0.142	0.356 ± 0.121

Values are expressed as mean ± standard deviation.

**Table 6:** Vitamin and mineral composition of the control and selected lamb pâté formulation (O-2).

Parameter	Control	O-2 (15% Offal)
Vitamin B1 (thiamine), mg/100 g	0.242 ± 0.048	0.257 ± 0.050
Vitamin B2 (riboflavin), mg/100 g	1.35 ± 0.56	1.64 ± 0.68
Vitamin B3 (niacin), mg/100 g	7.88 ± 1.58	7.90 ± 1.59
Vitamin B5 (pantothenic acid), mg/100 g	2.09 ± 0.42	2.30 ± 0.46
Iron (Fe), mg/100 g	7.80 ± 0.05 ^b^	8.11 ± 0.05 ^a^
Calcium (Ca), mg/100 g	34.73 ± 0.41 ^b^	38.01 ± 0.45 ^a^
Potassium (K), mg/100 g	368.10 ± 3.76 ^b^	379.45 ± 3.80 ^a^

Values are presented as mean ± SD. Different superscript letters within the same row, where indicated, denote significant differences between the control and O-2 formulations (*p* < 0.05).

**Table 7:** Microbiological safety and heavy metal assessment of lamb pâté enriched with 15% lamb offal.

Parameter	Regulatory Limit	O-2 (15% Offal)	Compliance
Total viable count (TVC), CFU/g	≤5 × 10^3^	2.1 × 10^2^	Complies
Coliform bacteria	Not detected in 1 g	Not detected	Complies
*Escherichia coli*	Not detected in 1 g	Not detected	Complies
*Staphylococcus aureus*	Not detected in 0.1 g	Not detected	Complies
*Salmonella* spp.	Not detected in 25 g	Not detected	Complies
Yeasts, CFU/cm^3^	Within permissible limits	11	Complies
Molds, CFU/cm^3^	Within permissible limits	3	Complies
Lead (Pb)	Not detected	Not detected	Complies
Mercury (Hg)	Not detected	Not detected	Complies

## Data Availability

The original contributions presented in this study are included in the article/Supplementary Materials. Further inquiries can be directed to the corresponding authors.

## Supplementary Materials

The following supporting information can be downloaded at: https://www.mdpi.com/article/10.3390/foods15203647/s1, Table S1: Individual replicate values for the physicochemical characteristics of the control and experimental lamb pâté formulations.

## Abbreviations

The following abbreviations are used in this manuscript:

WHC	Water-holding capacity
WRC	Water-retention capacity
a_w_	Water activity
TVC	Total viable count
