# Supplementary material for: Development of Functional Lamb Pâté Enriched with Edible Lamb By-Products and Pulse Ingredients: Physicochemical, Nutritional and Safety Evaluation
Source: Foods. 2026 Oct 14;15(20):3647. doi: 10.3390/foods15203647 (PMC13703730; doi:10.3390/foods15203647)
Supplement: Supplementary file 1 [file foods-15-03647-s001.zip › foods-4600558-supplementary.pdf]

Supplementary Materials

Table S1. Individual replicate values for the physicochemical characteristics of the control and experimental lamb pâté formulations.

Parameter	Sample	Rep 1	Rep 2	Rep 3	Mean ± SD
Protein (%)	Control	28.0	28.8	29.3	28.7 ± 0.66
Protein (%)	O-1	27.4	28.3	28.9	28.2 ± 0.75
Protein (%)	O-2	25.5	26.0	26.5	26.0 ± 0.50
Protein (%)	O-3	25.6	26.4	26.9	26.3 ± 0.65
Fat (%)	Control	25.0	25.9	26.5	25.8 ± 0.75
Fat (%)	O-1	22.7	23.4	23.8	23.3 ± 0.56
Fat (%)	O-2	24.2	24.9	25.6	24.9 ± 0.70
Fat (%)	O-3	22.6	23.3	24.0	23.3 ± 0.70
Moisture (%)	Control	60.7	61.8	62.6	61.7 ± 0.95
Moisture (%)	O-1	60.2	61.0	61.8	61.0 ± 0.80
Moisture (%)	O-2	61.6	62.5	63.4	62.5 ± 0.90
Moisture (%)	O-3	61.4	62.4	63.4	62.4 ± 1.00
pH	Control	5.71	5.80	5.86	5.79 ± 0.08
pH	O-1	4.83	4.89	4.95	4.89 ± 0.06
pH	O-2	4.85	4.90	4.95	4.90 ± 0.05
pH	O-3	4.83	4.90	4.97	4.90 ± 0.07
Water activity (a_x)	Control	0.9994	0.9997	1.0000	0.9997 ± 0.0003
Water activity (a_x)	O-1	0.9996	0.9998	1.0000	0.9998 ± 0.0002
Water activity (a_x)	O-2	0.9998	0.9999	1.0000	0.9999 ± 0.0001
Water activity (a_x)	O-3	0.9996	0.9998	1.0000	0.9998 ± 0.0002

Note: Values are individual analytical replicates (n = 3) and are presented together with the corresponding mean ± standard deviation (SD). The replicate values shown here correspond to the physicochemical data summarized in Table 2 of the main manuscript.
